# Health education for the prevention of hypertensive disorders of pregnancy

**DOI:** 10.31744/einstein_journal/2020CE6076

**Published:** 2020-11-12

**Authors:** Gustavo Gonçalves dos Santos

**Affiliations:** 1 Universidade Estadual Paulista “Júlio de Mesquita Filho” Faculdade de Medicina BotucatuSP Brazil Faculdade de Medicina, Universidade Estadual Paulista “Júlio de Mesquita Filho”, Botucatu, SP, Brazil.

Dear Editor,

I have some considerations about the article “Epidemiology of hypertension in pregnant women”.^(^^1^^)^ The study is strongly based on evidence scientific, shows that health education is important in this scenario, and demonstrates that inappropriate individual and collective may be associated to onset of hypertension. The following factors are related to hypertension: advanced age, family history, preexisting hypertension, diabetes, obesity and consumption of ultra-processed foods, and strongly corroborate the need for health education.^(^^2^^)^ Nurses and physicians are the first professionals to have contact with the pregnant woman and should early identify the signs of complications of hypertensive pregnancy syndromes, using tools that guide the essential actions, not only in the biological aspect of the disease, but also addressing the pregnant woman in her singularity.^(^^3^^–^^5^^)^

Congratulations to the authors! The issue must be fully discussed, and publishing on this topic is a path for excellence.
