# Supplementary material for: Health education for the prevention of hypertensive disorders of pregnancy
Source: Einstein (Sao Paulo). 2020 Nov 12;18:eCE6076. doi: 10.31744/einstein_journal/2020CE6076 (PMC7664823; doi:10.31744/einstein_journal/2020CE6076)
Supplement: Translation

# Translation: Educação em saúde para prevenção de síndromes hipertensivas da gestação

**Authors:** Gustavo Gonçalves dos Santos

**Affiliations:** 1 BotucatuSP Brasil Faculdade de Medicina, Universidade Estadual Paulista “Júlio de Mesquita Filho”, Botucatu, SP, Brasil.

Caro Editor,

Trago considerações acerca do artigo “Epidemiologia da hipertensão arterial em gestantes”.^(^^1^^)^O estudo, fortemente amparado por evidências científicas, mostra que se faz necessária a educação em saúde nesse cenário, além de evidenciar que hábitos individuais e coletivos inadequados podem estar associados ao surgimento da hipertensão arterial. Os fatores associados à hipertensão arterial idade elevada, antecedentes familiares, preexistência de hipertensão, diabetes, obesidade e consumo de alimentos ultraprocessados, corroboram a forte necessidade de educação em saúde.^(^^2^^)^Os enfermeiros e médicos são os primeiros profissionais a ter contato com a gestante e devem identificar, de maneira precoce, os sinais de complicações das síndromes hipertensivas da gestação, a partir de instrumentos que norteiem as ações essenciais, não apenas no aspecto biológico da doença, mas também contemplando a gestante em sua singularidade.^(^^3^^–^^5^^)^

Parabenizo os autores! A questão deve ser amplamente discutida, e a publicação sobre esse assunto é um caminho para excelência.
